# Effect of radio frequency heating on nutritional quality and protein solubility of corn

**DOI:** 10.1002/fsn3.332

**Published:** 2016-01-15

**Authors:** Amro B. Hassan, Elke Pawelzik, Dieter von Hoersten

**Affiliations:** ^1^ Department of Crop SciencesSection of Agricultural EngineeringFaculty of Agricultural ScienceGeorg‐August UniversityGoettingenGermany; ^2^ Environment and Natural Resource Desertification Research Institute (ENDRI)National Center for ResearchP.O. Box 6096KhartoumSudan; ^3^ Department of Crop SciencesDivision Quality of Plant ProductsGeorg‐August‐University of GoettingenCarl‐Sprengel‐Weg 1D‐37075GoettingenGermany; ^4^ Julius Kühn ‐ Institut (JKI)Federal Research Centre for Cultivated PlantsBraunschweigGermany

**Keywords:** Corn, nutritional quality, protein solubility, radio frequency

## Abstract

In this study, radio frequency heat treatment at varying temperatures (50, 55, and 60°C) was applied to investigate its impact on the nutritional quality and protein solubility of corn. The nutritive value was measured in terms of crude protein content, in vitro protein digestibility, bioavailability of Fe and Ca, and antinutritional factors, tannins and total polyphenols contents. No significant change in total and digestible protein of corn flour was observed after treatments. On the other hand, the availability of Ca and Fe was significantly increased, whereas the antinutritional factors, tannins and total polyphenols contents were decreased after radio frequency heating. Moreover, protein solubility was found significantly (*P* < 0.05) higher in treated corn than in control sample. Regarding these results, radio frequency heating at controlled temperature up to 60°C might be used as postharvest method to enhance the nutritional quality of corn.

## Introduction

Radio frequency (RF) (1–300 MHz) are non‐ionizing electromagnetic waves that generates heat volumetrically within dielectrical materials due to the polarization mechanisms of the dipole molecule rotation and direct conduction effect, which lead to rapid and uniform heating process (Metaxas and Meredith 1993; Piyasena et al. 2003). Radio frequency energy has been researched to control primary and secondary insects pest in agricultural products (Wang et al. 2001, 2002, 2004; Guo et al. 2011).

Besides controlling stored pests, radio frequency influenced the quality characteristics of agricultural products. Gao et al. (2010) investigated the influence of radio frequency heat treatment on in‐shell and shelled almond quality. They concluded that almond quality was not affected by the radio frequency treatment, however, the peroxide value, fatty acid, color, and moisture content of treated almond were found better than or similar to untreated one. Similar observation was also reported in radio frequency‐treated walnuts (Wang et al. 2001, 2002, 2004). However, few studies have examined the impact of radio frequency on the nutritional quality of grains. Therefore, the purpose of this study was to estimate the nutritional value and protein solubility of corn after radio frequency heating at different temperatures.

## Materials and Methods

### Construction of the radio frequency system

A 3 kW, 27.12 MHz radio frequency system (Sairem, S.A.S, 1511, France) was used in this study. This system consists of RF power generator with power capacity between 0 and 3 kW, matching box system, and applicator. The RF applicator includes a pair of radio frequency electrode 600 × 400 mm. During the treatments, the grain temperature was measured in three different locations using fiber optic thermometers.

### Samples preparation

Corn (*Zea mays* L.) cultivar “Amadeo” used in this study were obtained from KWS SAAT AG; Einbeck, Germany. The moisture content of the grains was equilibrated to 14% (w.b) then treated with radio frequency at 300 W, for different target temperatures 50, 55, and 60°C for 1 min. During the treatments, the grains were placed into a Teflon plate attached to three fiber optic sensors (FOB4, OMEGA, Germany). To measure grain temperature, the sensors were placed at three different locations in the plate. After treatments, corn flour was prepared using laboratory miller (Retsch, Germany). The milled samples were packaged in a polyethylene bag and kept into a desiccator at room temperature.

### Chemical analysis

Total protein of corn flour was measured by Dumas's method according to Sweeney and Rexroad (1987). The in vitro protein digestibility was determined according to the method of Maliwal (1983) as described by Monjula and John (1991). Quantitative estimation of tannin for each sample was carried out using modified‐vanillin‐HCl in methanol method as described by Price et al. (1978). Total polyphenols were determined according to the Prussian blue spectrophotometric method (Price and Butler 1977).

Mineral availability was determined after extracted by HCl (0.03 mol/L) according to the method described by Chauhan and Mahjan (1988). The available Ca was determined by a titration method and Fe was determined by atomic absorption spectrophotometer (AOAC 1995).

Protein solubility was determined by the procedure of Hagenmaier (1972) with a slight modification. Flour was suspended in distilled water, the suspension was thoroughly mixed and centrifuged at 1038 *g* for 20 min at room temperature. The soluble nitrogen in the supernatant was estimated by the micro‐Kjeldahl assays (AOAC 1995).

### Statistical analysis

For each temperature, radio frequency treatment was applied three times, and then each sample was analyzed in triplicate and the values were then averaged. Data were analyzed using analysis of variance (ANOVA). Significant differences were calculated (*P* < 0.05) using least significant difference (LSD).

## Results and Discussion

### Effect of radio frequency heating on total protein and in vitro protein digestibility

**Figure 1:**
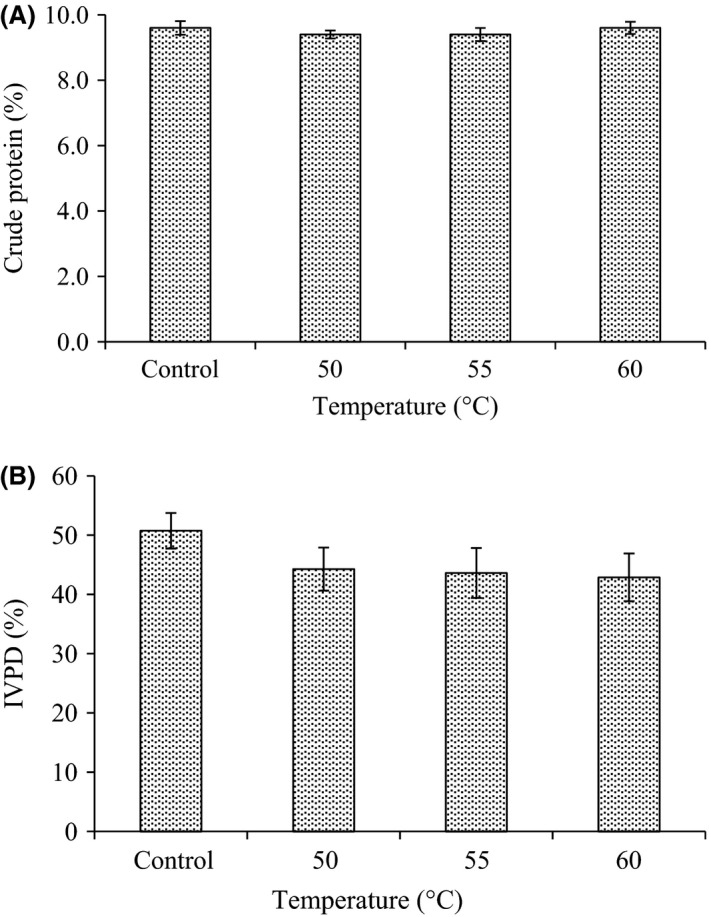
Effect of radio frequency heating on (A) crude protein content and (B) in vitro protein digestibility (IVPD) in corn. Values are means (±SD).

Before the treatment, the total protein of corn was found to be 9.6% (Fig.1A). The analysis of variance showed that radio frequency heat treatment at temperature up to 60°C caused insignificant change in total protein of corn flour. As shown in Figure 1B, the in vitro protein digestibility (IVPD) of untreated sample was found to be 50.8%. Radio frequency heating caused a minor reduction on the IVPD to 44.3, 43.3, and 42.9% at the temperatures 50, 55, and 60°C, respectively. The reduction in IVPD resulting from heat processing of grain might be due to the formation of complexes between proteins and other grain components during treatments. Moreover, the reduction in protein digestibility of corn might be associated with amino acid degradation and Maillard reaction during heat processing (Hurrell et al. 1976; Hsu et al. 1977; Nestares et al. 1993).

### Effect of radio frequency heating on tannins and total polyphenol content

**Figure 2:**
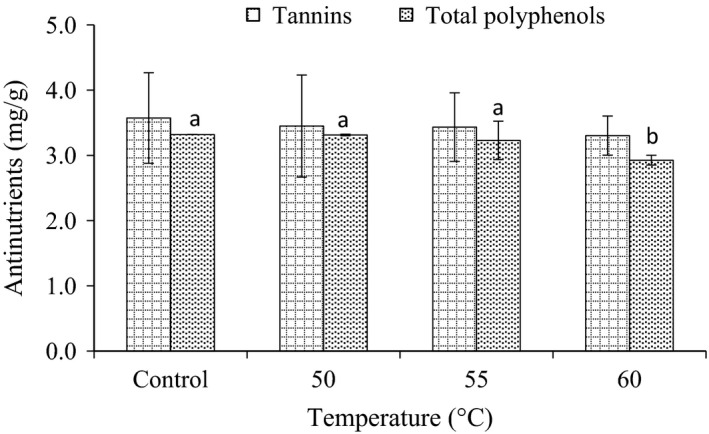
Effect of radio frequency heating on tannins and total polyphenols contents in corn. Values are means (±SD). Values not sharing a common superscript are significantly (*P* < 0.05) different.

As illustrated in Figure 2, the tannin content of corn was found to be 3.6 mg/g prior to radio frequency heating. Tannin content of the investigated samples showed a dose‐dependent decrease. The reduction rate of tannin content was found to be 2.8, 2.6, and 8.3% when corn were treated at temperature of 50, 55, and 60°C, respectively, compared to control one.

On the other hand, the total polyphenols of untreated corn was found to be 3.3 mg/g. Radio frequency heating of the seeds at 60°C significantly (*P* < 0.05) reduced the polyphenol content to 2.9 mg/g.

Radio frequency heating causes a reduction in antinutritional factors such as tannin and total polyphenols content. This could be due to decomposition of phenols or formation of their complexes with protein during heating. Similarly, the obtained results agree with observation reported by Wang et al. (2008) who stated that application of dielectric heating by microwave results in significant reduction in antinutritional factors in pulses.

### Effect of radio frequency heating on available Fe and Ca

**Figure 3:**
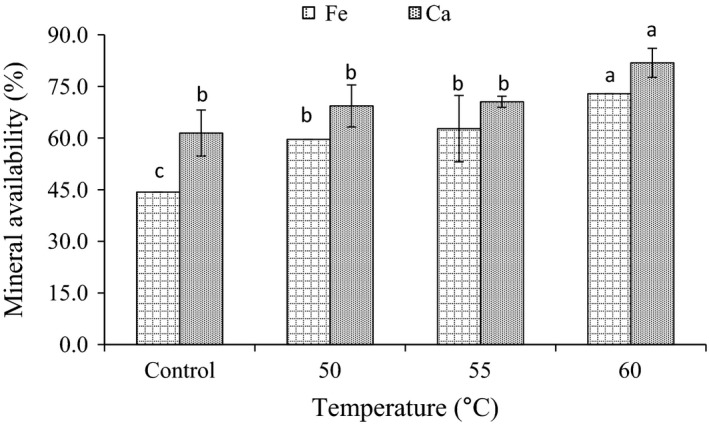
Effect of radio frequency heating on available Ca and Fe in corn. Values are means (±SD). Values not sharing a common superscript are significantly (*P* < 0.05) different.

The availability of Fe and Ca was found to be 44.3 and 61.5%, respectively, before the treatments as shown in Figure 3. Radio frequency heating of corn increased the availability of Fe and Ca. It was increased as the temperature was increased. Heating of the seeds at 60°C was showed the highest values of the availability, which were found 72.9 and 81.9% for Fe and Ca, respectively. Increasing of available minerals after radio frequency heating might be result from the reduction in the amount of antinutritional factors such as tannins and polyphenols.

### Effect of radio frequency heating on protein solubility

**Figure 4:**
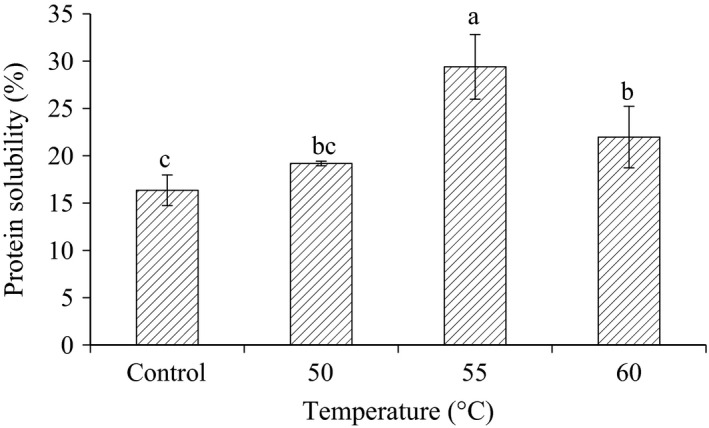
Effect of radio frequency heating on protein solubility in corn. Values are means (±SD). Values not sharing a common superscript are significantly (*P* < 0.05) different.

Among the functional properties of proteins, solubility is probably the most critical function. As shown in Figure 4, the protein solubility of raw corn was found to be 16.3%. Treatment of corn with radio frequency caused a significant (*P* < 0.05) increment in protein solubility. Increment of protein solubility after heating might be due to the high‐proteolytic activity during treatment, which may lead to hydrolysis of the stored proteins.

Although there was a significant increase in the solubility of the protein after treatment, it was clearly observed that the percentage of protein solubility start to decrease after the temperature of 55°C, particularly at 60°C. This phenomenon might be explained by the effect of heating which resulting in the denaturation of the protein.

## Conclusion

Results of the present investigation tend to suggest that radio frequency heat treatments of corn under controlled temperature enhanced the nutritional value of corn, by reducing its antinutritional factors and increased in the availability of some minerals. On the other hand, radio frequency treatments improve the protein solubility of corn. Hence, it can be used as an effective postharvest method to improve the quality of products. However, further researches are needed to investigate the effect of several factors such as moisture content levels, different varieties of agriculture commodities and application times on radio frequency technology to improve its efficiency to reach the reasonable usage in future.

## Conflict of Interest

None declared.
